# Epidemiology and risk factors of extensively drug-resistant *Pseudomonas aeruginosa* infections

**DOI:** 10.1371/journal.pone.0193431

**Published:** 2018-02-22

**Authors:** Nattawan Palavutitotai, Anupop Jitmuang, Sasima Tongsai, Pattarachai Kiratisin, Nasikarn Angkasekwinai

**Affiliations:** 1 Division of Infectious Diseases and Tropical Medicine, Department of Medicine, Faculty of Medicine, Siriraj Hospital, Mahidol University, Bangkok, Thailand; 2 Department of Microbiology, Faculty of Medicine, Siriraj Hospital, Mahidol University, Bangkok, Thailand; Emory University School of Medicine, UNITED STATES

## Abstract

**Background:**

The incidence of nosocomial infections from extensively drug-resistant *Pseudomonas aeruginosa* (XDR-PA) has been increasing worldwide. We investigated the prevalence and factors associated with XDR-PA infections, including the factors that predict mortality.

**Methods:**

We retrospectively studied a cohort of adult, hospitalized patients with *P*. *aeruginosa* (PA) infections between April and December 2014.

**Results:**

Of the 255 patients with PA infections, 56 (22%) were due to XDR-PA, 32 (12.5%) to multidrug resistant *Pseudomonas aeruginosa* (MDR-PA), and 167 (65.5%) to non-MDR PA. Receiving total parenteral nutrition (adjusted OR [aOR] 6.21; 95% CI 1.05–36.70), prior carbapenem use (aOR 4.88; 95% CI 2.36–10.08), and prior fluoroquinolone use (aOR 3.38; 95% CI 1.44–7.97) were independently associated with the XDR-PA infections. All XDR-PA remained susceptible to colistin. Factors associated with mortality attributable to the infections were the presence of sepsis/septic shock (aOR 11.60; 95% CI 4.66–28.82), admission to a medical department (aOR 4.67; 95% CI 1.81–12.06), receiving a central venous catheter (aOR 3.78; 95% CI 1.50–9.57), and XDR-PA infection (aOR 2.73; 95% CI 1.05–7.08).

**Conclusion:**

The prevalence of XDR-PA infections represented almost a quarter of *Pseudomonas aeruginosa* hospital-acquired infections and rendered a higher mortality. The prompt administration of an appropriate empirical antibiotic should be considered when an XDR-PA infection is suspected.

## Introduction

*Pseudomonas aeruginosa* (PA) has become an important cause of healthcare-associated infections (HAIs) [1, 2]. Carbapenem has been widely used for empirical or directed therapy when a PA infection is suspected due to its natural resistance against several antibiotics [3]. However, recent data from the National Antimicrobial Resistance Surveillance, Thailand (NARST), revealed an increasing trend of carbapenem-resistant *P*. *aeruginosa* (CRPA), from approximately 15% of infections in 2000–2005 to 30% in 2009–2013 [4, 5]. A study conducted in eight tertiary hospitals across Thailand during 2007–2009 also found that 72% of 261 multidrug-resistant *P*. *aeruginosa* (MDR-PA) isolates were CRPA [6]. Moreover, the recent emergence of extensively drug-resistant *P*. *aeruginosa* (XDR-PA), which is defined as those strains that remain susceptible to only one or two classes of antipseudomonal agents, has become a serious concern due to the lack of an effective antimicrobial therapy [7, 8].

Inappropriate initial antimicrobial treatment for CRPA and XDR-PA infections may lead to unfavorable outcomes [9–11]. Knowing the epidemiology and potential risk factors of XDR-PA infections is crucial for the early selection of suitable antimicrobial agents. However, previous studies of the prevalence and risk factors for XDR-PA infections or acquisition focused only on specific populations, such as patients with cancer [12], hematologic malignancies [13], solid organ transplant recipients [14] or patients with XDR-PA bacteremia [15–17]. Therefore, the results might not be applicable to all patient populations. This study aimed to determine the prevalence and factors associated with XDR-PA infections and their outcomes in general healthcare settings.

## Materials and methods

### Patients and data collection

We conducted a retrospective cohort study at Siriraj Hospital, a 2,200-bed, tertiary-care, university hospital in Bangkok, Thailand, between April and December 2014. The protocol for this study was approved by the Scientific Ethics Committee of the Siriraj Institutional Review Board (IRB). Waiver of consent was approved by the IRB, and data was anonymized prior to analysis. Eligible patients included all hospitalized adults, aged ≥18 years, who had suffered HAIs, as defined by the Centers for Disease Control and Prevention (CDC), [18, 19] from PA. The study also included PA with polymicrobial co-infections. Patients who were the subjects of end-of-life decisions were excluded.

Patient information was derived from medical records via retrospective review. Demographic and other relevant data, including treatment outcomes, were recorded. Clinical outcomes were assessed at 7 days after receiving treatment and at the end of therapy. Mortality was assessed at 28 days after completing treatment. To assess the severity of illness, APACHE II scores [20] were measured within 24 hours of admission for patients with PA bacteremia and for those who were admitted to the intensive care unit (ICU).

#### Definitions

PA from patient samples was identified by cultures on standard media, including biochemical testing. Prior PA colonization was defined as the isolation of PA from any clinical specimens in the absence of signs or symptoms of infections, and the patients had not received antimicrobial treatment related to the culture results. Prior antimicrobial therapy was defined as any exposure to an antibiotic for at least 48 hours within the preceding 90 days. Antimicrobial testing of susceptibility to the antipseudomonal antimicrobial drug categories (aminoglycosides, antipseudomonal carbapenems, antipseudomonal cephalosporins, antipseudomonal fluoroquinolones, antipseudomonal penicillins, and ß-lactamase inhibitors) and colistin were performed by the disc diffusion method, according to the Clinical and Laboratory Standards Institute 2010 [21].

Based on the antimicrobial susceptibility testing (AST) results, PA were classified using the most-current standard definitions [22], as follows: (a) non-MDR PA: a strain that does not fit the definition of MDR-PA; (b) MDR-PA: a strain not-susceptible to at least one agent in three or more antipseudomonal antimicrobial categories; (c) XDR-PA: a strain not-susceptible to at least one agent in all but two or fewer antipseudomonal antimicrobial categories; and (d) PDR-PA: strains not-susceptible to all agents in all antipseudomonal antimicrobial categories. Strains with intermediate susceptibility to antimicrobial agents were considered resistant. The non-MDR PA and MDR-PA isolates were included in the non-XDR PA group, as opposed to the XDR-PA group.

An appropriate empirical antimicrobial therapy was deemed to be an appropriate dosage regimen of antibiotics to which the organisms were sensitive *in vitro*, prescribed within 24 hours of the infection onset. An effective definitive antimicrobial therapy was defined as a treatment using at least one effective antipseudomonal agent, based on the AST results. The length of stay (LOS) was defined as the length of inpatient episode from the day of admission to the day of discharge.

#### Patient outcomes

Clinical outcomes were assessed at 7 days after the onset of infection and at the end of therapy. The clinical outcomes were defined as follows: cured (complete resolution of fever, clinical signs and other relevant investigations); improved (partial resolution of fever, clinical signs and other relevant investigations); stable (no change in clinical signs and other relevant investigations); worsened (worsened fever, clinical signs and other relevant investigations); death; and unknown. Favorable clinical outcomes comprised cured and improved status, whereas stable, worsened status and death were classified as unfavorable clinical outcomes.

Microbiological responses were assessed at 7 days and at the end of therapy. The microbiological outcomes were defined as follows: eradicated (negative culture of PA following a course of antibiotic therapy); persistent (persistent isolation at that site of infection); new infection (found new organisms at that site of infection); and indeterminate (absence of subsequent cultures to assess microbiological response). The favorable microbiological outcome was eradicated, while the unfavorable microbiological outcomes were persistent or new infections.

Mortality was assessed at 28 days after patients completed treatment. It was classified as: (a) mortality directly attributable to infection; i.e., death with clinical evidence of an active infection (elevated white blood cell count, temperature or systolic blood pressure <90 mmHg); (b) mortality unrelated to infection; i.e., death occurring during hospitalization after an episode of infection but due to causes independent of the infectious process; (c) survived; and (d) unknown [23].

### Sample size calculation

Our main objective was to determine the prevalence of extensively-drug resistant *P*. *aeruginosa* (XDR-PA) in a general health care setting. As we did not know the prevalence of XDR-PA in our setting, the prevalence of CRPA was used to calculate the required sample size to estimate a single proportion. Based on surveillance data from our institute in 2012, the prevalence of HAIs caused by CRPA was 30%; this rate was used to calculate the sample size of the study. Using NQuery Advisor version 5.0 to determine the prevalence of XDR-PA infections within 5% with a 95% level of confidence, a necessary evaluable sample size of 323 patients was calculated. As we estimated a non-evaluable rate of 20%, an enrollment of 388 patients was required.

In addition, we wanted to examine the factors associated with the infections caused by XDR-PA. A variety of exposures or factors could influence the risk of developing such infections, including a comorbid disease or immune status, prior antibiotic use, prior colonization or infection with *P*. *aeruginosa*, length of stay, prior *P*. *aeruginosa* infection, the site of the infection, the receiving of a medical device, or even the severity of the illness. Therefore, to control multiple confounders and ensure the true association of the various factors with the outcomes, we adjusted for potential confounders by using multivariate regression to identify the independent factors associated with XDR-PA infections and those associated with mortality attributable to the infections.

To calculate the sample size for a multiple logistic regression model which would be used to identify the factors associated with mortality, we assumed that the mortality rate of patients in the non-XDR-PA group was 20%, with an approximately moderate effect size (odd ratio) of 0.3, and assumed that the factors in the model have a squared multiple correlation with XDR-PA of 0.09. Using nQuery Advisor with a two-sided significance level of 0.05 and a power of 80%, the required sample size was calculated to be 218 participants.

### Statistical analysis

We produced descriptive statistics for the patients’ demographic and baseline clinical characteristics. Continuous data were expressed as mean and standard deviations (SD) or median and range, whereas categorical data were expressed as numbers and percentages. To compare the baseline characteristics for the XDR-PA and non-XDR PA infections, either an unpaired *t*-test or the Mann–Whitney U test was used for the continuous data, and either the chi-square or Fisher’s exact test for the categorical data, as appropriate. Multiple logistic regression analysis was used to identify the independent factors related to XDR-PA infection and mortality attributable to infection. Age and the factors for which *P*< 0.1 in the univariate analysis were selected for the backward multiple logistic regression analysis. All factors for which *P*>0.10 were removed from the final model. The analyses were performed using PASW Statistics version 18 (SPSS Inc., Chicago, IL, USA). All tests were two-sided. *P*<0.05 was considered significant.

## Results

### Demographic and clinical characteristics

A total of 255 patients with *P*. *aeruginosa* infections were included during the 9-month study period. Of those, 56 (22%) strains were XDR-PA, 32 (12.5%) were MDR-PA, and 167 (65.5%) were non-MDR PA. Among the 101 (39.6%) CRPA strains, 54 (53.5%) were XDR-PA, 25 (24.8%) were MDR-PA, and 22 (21.8%) were non-MDR PA. This study found no PDR-PA strains.

**Fig 1:**
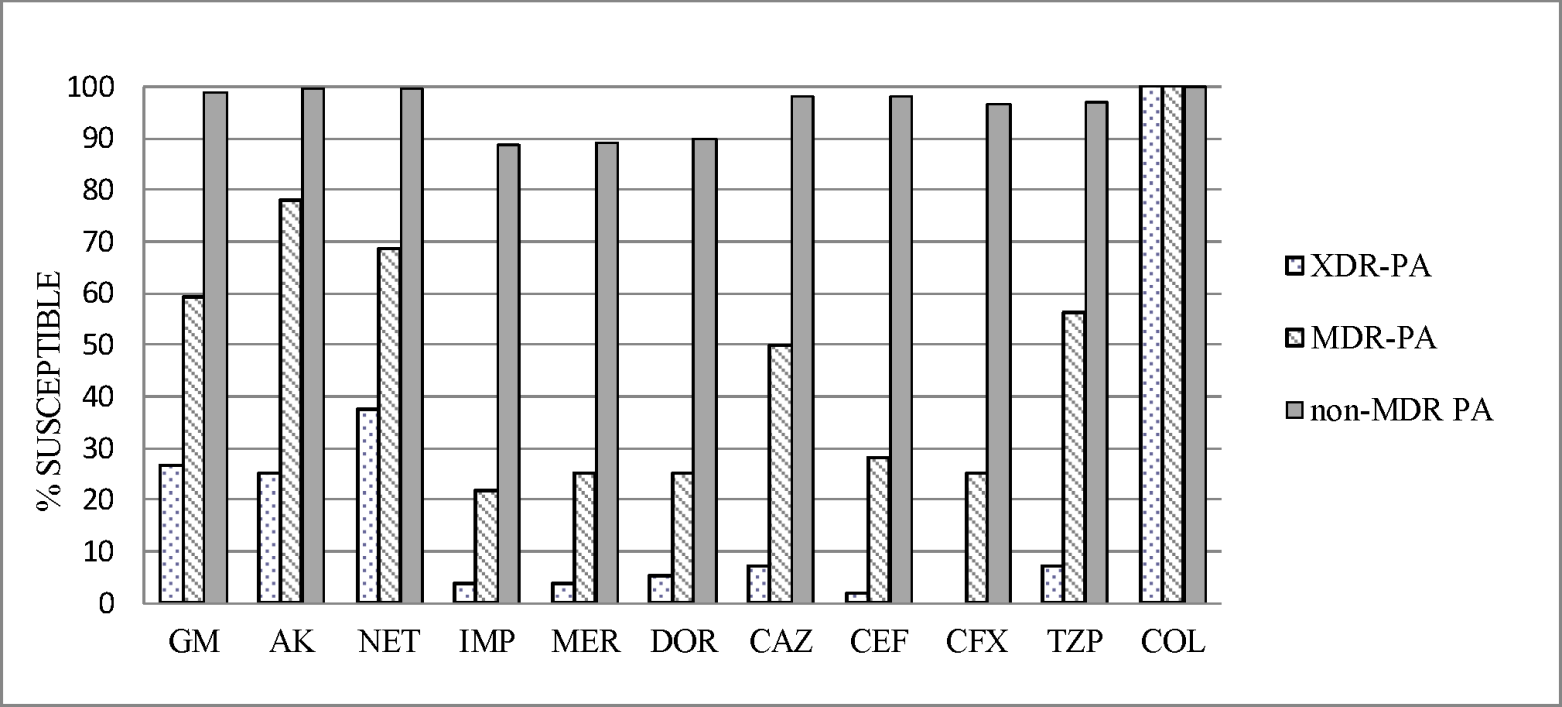
Rate (%) of antimicrobial susceptibility to 11 antipseudomonal antimicrobial agents of extensively drug-resistant *Pseudomonas aeruginosa* (XDR-PA), multidrug resistant *P*. *aeruginosa* (MDR-PA), and non-MDR PA. AK: amikacin; CAZ: ceftazidime; CEF: cefepime; CFX: ciprofloxacin; COL: colistin; DOR: doripenem; GM: gentamicin; IMP: imipenem; MER: meropenem; NET: netilmicin; TZP: Piperacillin/tazobactam.

Most patients (57.3%) were male, with a mean age of 64.9 (±17.6) years. Approximately 90% of patients had underlying diseases. The clinical characteristics of patients with XDR-PA and non-XDR PA infections are at Table 1. The rates of antimicrobial susceptibility for the 11 anti-pseudomonal agents in the study are at Fig 1. Anonymous dataset has also been provided within supporting information file (S1 Dataset).

**Table 1:** Demographics and clinical characteristics of patients with extensively drug-resistant *Pseudomonas aeruginosa* (XDR-PA) and non-XDR-PA infection.

Factors	XDR-PA	Non-XDR PA^#^	*P*	
	n = 56	n = 199		
Mean age ± SD (years)	68.0±15.1	64.0±18.2	0.20	
Median (range)	68.0 (18–96)	66.0 (18–105)		
Male, *n* (%)	34 (60.7)	112 (56.3)	0.66	
Medical admission *n* (%)	27 (48.2)	110 (55.3)	0.43	
ICU admission, *n* (%)	8 (14.3)	42 (21.1)	0.34	
Underlying diseases, *n* (%)	53 (94.6)	177 (88.9)	0.31	
Hypertension	35 (62.5)	122 (61.3)	0.99	
Chronic kidney disease	23 (41.1)	56 (28.1)	0.09	
Diabetes mellitus	21 (37.5)	66 (33.2)	0.66	
Heart disease	15 (26.8)	58 (29.1)	0.86	
Solid malignancy	14 (25.0)	49 (24.6)	1.00	
Cerebrovascular disease	13 (23.2)	71 (35.7)	0.11	
Chronic lung disease	8 (14.3)	24 (12.1)	0.83	
Other hematologic diseases	5 (8.9)	17 (8.5)	1.00	
Hematologic malignancy	5 (8.9)	23 (11.6)	0.75	
On immunosuppressive agents	5 (8.9)	29 (14.6)	0.38	
Cirrhosis	5 (8.9)	11 (5.5)	0.54	
Neutropenia	3 (5.4)	14 (7.0)	1.00	
Mean length of stay ± SD (days)	53.5±40.1	45.5±40.1	0.09	
Median (range)	42 (8–162)	34 (7–252)		
Prior ICU stays within 3 months, *n* (%)	11 (19.6)	15 (7.5)	0.02	
Prior *P aeruginosa* colonization, *n* (%)	12 (21.4)	25 (12.6)	0.15	
Prior *P aeruginosa* infections, *n* (%)	9 (16.1)	16 (8.0)	0.13	
Prior antimicrobial agents use, *n* (%)	54 (96.4)	176 (88.4)	0.13	
Carbapenem	44 (78.6)	74 (37.2)	<0.001
Carbapenem used within 2 weeks	38 (67.9)	60 (30.2)	<0.001
3^rd^ or 4^th^ generation cephalosporin	28 (50.0)	79 (39.7)	0.22
Fluoroquinolone	15 (26.8)	17 (8.5)	0.001
Vancomycin	13 (23.2)	25 (12.6)	0.08
Colistin	7 (12.5)	19 (9.5)	0.69
Mean LOS prior to *P aeruginosa* HAIs ±SD (days)	28.5±28.7	19.6±27.5	0.03
Median (range)	17.5 (2–147)	10 (2–180)	
Receiving invasive medical device, *n* (%)	49 (87.5)	155 (77.9)	0.16
Foley catheter	42 (75.0)	134 (67.3)	0.35
Nasogastric tube	36 (64.3)	118 (59.3)	0.60
Mechanical ventilation	19 (33.9)	72 (36.2)	0.88
Central venous catheter	19 (33.9)	38 (19.1)	0.03
Total parenteral nutrition	5 (8.9)	2 (1.0)	0.006
Type of infection, *n* (%)			
Polymicrobial infection	44 (78.6)	122 (61.3)	0.03
Monomicrobial infection	12 (21.4)	77 (38.7)	
Sepsis/septic shock, *n* (%)	14 (25.0)	35 (17.6)	0.29
APACHE II scores*, *n*	14	76	
Mean ± SD	11.1±4.9	10.2 ±5.1	0.54
Sites of infections, *n* (%)			
Late onset VAP	12 (21.4)	23 (11.6)	0.09
CA-UTI	13 (23.2)	30 (15.1)	0.22
Late onset HAP	7 (12.5)	32 (16.1)	0.66
Skin & soft tissue infection	5 (8.9)	16 (8.0)	0.79
GI infection	5 (8.9)	7 (3.5)	0.14
LRTI	3 (5.4)	15 (7.5)	0.77
Primary bloodstream infection	3 (5.4)	17 (8.5)	0.71
UTI	3 (5.4)	8 (4.0)	0.95
CRBSI	0	6 (3.0)	0.34
Surgical site infection	1 (1.8)	4 (2.0)	1.00
Early onset HAP	0	27 (13.6)	0.008
Early onset VAP	0	10 (5.0)	0.12

APACHE: Acute Physiology and Chronic Health Evaluation; BSI: bloodstream infection; CA-UTI: catheter-associated urinary tract infection; CRBSI: catheter-related bloodstream infection; HAIs: hospital-acquired infections; HAP: hospital-acquired pneumonia; ICU: intensive care unit; SD: standard deviation; VAP: ventilator associated pneumonia; LOS: length of stay

^#^ Non-XDR-PA included MDR-PA and non-MDR PA

* APACHE II score and sepsis/ septic shock were assessed within 24 hours of admission.

### Treatment and outcomes

The treatments and the clinical and microbiological outcomes of the PA infections are at Table 2. Patients in the XDR-PA group received appropriate empirical antimicrobial therapy less often than those in the non-XDR PA group (37.5% vs. 68.8%, *P*<0.001). Empirical therapy with a single antibiotic (particularly carbapenem) was frequently prescribed in both groups. Empirical combination antibiotics were more frequently used in the XDR-PA group (20.8% vs. 9.1%, *P* = 0.04); colistin plus carbapenem or piperacillin/tazobactam were the most commonly prescribed regimens in the XDR-PA group.

**Table 2:** Treatment, and clinical and microbiological outcomes, of patients with *Pseudomonas aeruginosa* infections.

Factors	XDR-PA	Non-XDR-PA	*P*
	n = 56	n = 199	
Received empirical antibiotic, *n* (%)	54 (96.4)	186 (93.5)	0.53
Received appropriate empirical antibiotic, *n* (%)	21 (37.5)	137 (68.8)	<0.001
Type of empirical antibiotics, *n*			
Single antibiotics	42 (79.2)	169 (90.9)	0.04
Carbapenem	26 (61.9)	70 (41.4)	
Piperacillin/tazobactam	6 (14.2)	54 (32.0)	
Colistin	4 (9.5)	6 (3.5)	
Cefepime or ceftazidime	2 (4.8)	30 (17.8)	
Fluoroquinolone	2 (4.8)	7 (4.1)	
Aminoglycoside	2 (4.8)	0	
Fosfomycin	0	2 (1.2)	
Combination antibiotics	11 (20.8)	17 (9.1)	0.04
Colistin + carbapenem	4 (36.4)	3 (17.6)	
Colistin + piperacillin/tazobactam	2 (18.2)	1 (5.9)	
Cefepime or ceftazidime + fluoroquinolone	2 (18.2)	0	
Colistin + cefepime or ceftazidime	1 (9.1)	0	
Carbapenem + fluoroquinolone	0	5 (29.4)	
Other combination regimen*	2 (18.2)	8 (47.1)	
Received definitive therapy, *n* (%)	56 (100.0)	197 (99.0)	1.00
Received effective definitive therapy, *n* (%)	45 (80.4)	196 (98.5)	<0.001
Type of definitive therapy, *n*			
Single antibiotics, *n* (%)	33 (58.9)	170 (85.4)	<0.001
Colistin	17 (51.5)	6 (3.5)	
Piperacillin/tazobactam	5 (15.2)	52 (30.6)	
Fluoroquinolone	0	19 (11.2)	
Aminoglycoside	1 (3.0)	0	
Carbapenem	10 (30.3)	49 (28.8)	
Cefepime or ceftazidime	0	41 (24.1)	
Fosfomycin	0	1 (0.6)	
Others	0	2 (1.2)	
Combination antibiotics, n (%)	23 (41.1)	29 (14.6)	<0.001
Colistin + carbapenem	15 (26.8)	7 (3.5)	
Colistin + piperacillin/tazobactam	2 (3.6)	2 (1.0)	
Colistin + fosfomycin	2 (3.6)	2 (1.0)	
Colistin + fluoroquinolone	2 (3.6)	1 (0.5)	
Cefepime or ceftazidime + fluoroquinolone	0	4 (2.0)	
Other combination regimens^$^	2 (3.6)	13 (44.8)	
Favorable clinical outcome, *n* (%)			
At 7 days	33 (58.9)	164 (82.4)	<0.001
At end of treatment	35 (62.5)	150 (75.4)	0.08
Favorable microbiological outcome, *n* (%)			
At 7 days	n = 46	n = 140	
	12 (26.1)	62 (44.3)	0.04
At end of treatment	n = 37	n = 123	
	10 (27.0)	60 (48.8)	0.03
Overall in-hospital mortality, *n* (%)	23 (41.1)	50 (25.1)	0.03
Mortality attributable to infection, *n* (%)	21 (37.5)	34 (17.1)	0.002

*n*: number; SD: standard deviation; XDR-PA: extensively drug-resistant *Pseudomonas aeruginosa*.

*Other combination regimen: 2 in XDR group received carbapenem plus vancomycin and colistin plus aminoglycoside; in non-XDR group (n = 8): 2 received carbapenem plus vancomycin, 2 received carbapenem plus fosfomycin, 2 received antipseudomonal penicillins and ß-lactamase inhibitors (BLBI) plus aminoglycoside, 1 received BLBI plus fluoroquinolone, and 1 received cephalosporin plus aminoglycoside.

^$^Other definitive combination regimen: 2 in XDR group received cephalosporin plus fosfomycin and colistin plus aminoglycoside; in non-XDR group (n = 13): 3 received BLBI plus fluoroquinolone, 2 received BLBI plus aminoglycoside, 2 received carbapenem plus aminoglycoside, 2 received cephalosporin plus fluoroquinolone, 2 received fluoroquinolone plus fosfomycin, 1 received carbapenem plus fosfomycin, and 1 received fluoroquinolone plus other antibiotic.

Infection with XDR-PA was associated with decreased favorable clinical outcomes at 7 days (*P*<0.001), as well as with delayed microbiological eradication at 7 days (*P* = 0.04) and at the end of therapy (*P* = 0.03). The overall mortality rate in this study was 34.5%, and it was significantly higher in the XDR-PA group than the non-XDR-PA group (41.1% vs. 25.1%, *P* = 0.03), including infection-related deaths (37.5% vs. 17.1%, *P* = 0.002).

### Factors associated with XDR-PA infections and the outcomes

The multivariable analysis showed that receiving total parenteral nutrition, prior carbapenem use, and prior fluoroquinolone use were independently associated with XDR-PA infections (Table 3). Patients with sepsis/septic shock, admission to a medical ward, receiving central venous catheter, and having an infection caused by XDR-PA were independently associated with the mortality attributable to infection (Table 4).

**Table 3:** Independent predictors of XDR-PA infections.

Factor	Crude OR (95% CI)	*P*	Adjusted OR^a^ (95% CI)	*P*
Total parenteral nutrition	9.66 (1.82–51.22)	0.008	6.21 (1.05–36.70)	0.044
Prior carbapenem use	6.19 (3.08–12.47)	<0.001	4.88 (2.36–10.08)	<0.001
Prior fluoroquinolone use	3.92 (1.81–8.48)	0.001	3.38 (1.44–7.97)	0.005

OR: odds ratio; XDR-PA: extensively drug-resistant *Pseudomonas aeruginosa*.

^a^Analysis adjusted for all factors listed plus age, chronic kidney disease, prior ICU stay within 3 months, carbapenem usage within 2 weeks, vancomycin usage, LOS prior to *P aeruginosa* HAIs, central venous catheter, polymicrobial infection, late-onset VAP and early-onset HAP. The 3 factors listed in this table were retained in the final model.

**Table 4:** Independent predictors of mortality attributable to infection.

Factor	Crude OR (95% CI)	*P*	Adjusted OR^a^ (95% CI)	*P*
Sepsis/septic shock	9.36 (4.52–19.37)	<0.001	11.60 (4.66–28.82)	<0.001
Medical admission	2.61 (1.37–4.96)	0.003	4.67 (1.81–12.06)	0.001
Central venous catheter	6.35 (3.21–12.54)	<0.001	3.78 (1.50–9.57)	0.005
Extensively drug-resistant	2.79 (1.44–5.41)	0.002	2.73 (1.05–7.08)	0.039
*Pseudomonas aeruginosa*				

OR: odds ratio

^a^Analysis adjusted for all factors listed, plus age, chronic kidney disease, prior ICU stays within 3 months, ICU admission, duration of hospitalization prior to *P*. *aeruginosa* infection, total parenteral nutrition, GI infection, CA-UTI, early onset HAP, late onset VAP, polymicrobial infection, received empirical antibiotic, and type of definite therapy. The 4 factors listed in this table were retained in the final model.

## Discussion

Hospital-acquired infections (HAI) caused by extensively drug-resistant (XDR) *P*. *aeruginosa* remain a therapeutic challenge as effective antimicrobial therapy is severely limited. In the present study, 22% of *P*. *aeruginosa* HAIs exhibited an XDR phenotype. A previous Thai study which focused on patients in a surgical intensive care unit identified that the prevalence of VAP caused by XDR-PA was only 1.7% [24]. However, the rate of XDR-PA infection for a general health care setting found in our study was equal to that observed in cancer patients with various sites of *P*. *aeruginosa* infection [12]. However, it was much lower than that reported for solid organ transplant recipients, namely, that 63% of *P*. *aeruginosa* bacteremia exhibited an XDR-PA phenotype [14]. In addition, almost 40% of *P*. *aeruginosa* HAIs in our study exhibited carbapenem resistance. Previous data for HAI reported to the National Healthcare Safety Network showed a lower prevalence of CRPA, ranging from 7% to 28% among 4,669 *P*. *aeruginosa* isolates collected from 4,515 hospitals in the U.S.A. between 2011 and 2014 [25]. Those results contrasted with the findings of a study in Thailand, in which Khuntayaporn and colleagues showed a high prevalence of CRPA (72%) among 261 isolates of MDR *P*. *aeruginosa* collected from eight tertiary hospitals across Thailand [6]. It should be noted that the prevalence of drug resistant *P*. *aeruginosa* may vary considerably between studies, depending on the study population and the site of isolation.

A high resistance rate of *P*. *aeruginosa* infections to carbapenem makes the provision of an appropriate empirical therapy challenging. According to the present study, colistin seems to be the only active agent for combating infections caused by XDR-PA. One approach for the treatment of infections caused by XDR pathogens is to combine several antibiotics with colistin in order to exert a synergistic effect or to decrease the possible toxicities associated with each drug [26]. Our study found that 20%–40% of XDR-PA strains remained sensitive to amikacin or netilmicin, but the combination of colistin and aminoglycosides probably increases renal toxicity, particularly in critically ill patients. Even though previous *in vitro* studies have shown a synergistic activity of imipenem, doripenem [27], ceftazidime or amikacin with colistin against MDR-PA or XDR-PA[28–32], there are no well-designed clinical studies establishing the superiority of this strategy over colistin monotherapy for the treatment of XDR-PA infections.

The factors contributing to the risk for XDR-PA infections are likely to vary with different patient populations. Our results are consistent with previous studies evaluating the potential risk for XDR-PA infections in patients with cancer and hematologic malignancy [12, 13]. Prior use of fluoroquinolone and carbapenem were found to be independently associated with XDR-PA infections. It is probably due to the antipseudomonal activity of these agents, which poses a greater tendency to select a resistant mutant than other antibiotics. Our study also found an independent association between total parenteral nutrition and XDR-PA infections. Similar to previous findings, parenteral nutrition was shown to be associated with the acquisition of MDRPA [33] or bacteremia due to CRPA [34]. However, in contrast to previous findings, we found no association of either previous PA colonization or high APACHE II scores with XDR-PA infections [14, 16, 17]. Since the surveillance culture is not routinely performed in our hospital, we probably underestimated the association of a previous PA colonization with XDR-PA infections.

Unsurprisingly, mortality with XDR-PA in this study was high; 41% of patients had fatal outcomes, which was similar to that observed in a previous study of cancer patients [12]. Consistent with that study, an infection due to XDR-PA isolates was associated with mortality. In addition, the presence of sepsis/septic shock, admission to a medical ward, and having a central venous catheter were independent factors associated with mortality attributable to the infections. Similar to a previous report examining the predictors of mortality in ventilator-associated pneumonia (VAP) caused by MDR-PA and XDR-PA, multiorgan dysfunction syndrome (MOD) was found to be associated with mortality [35]. This could be explained by the fact that severely ill patients who frequently require invasive medical devices are more susceptible to subsequent, recurrent infections, and to death due to infectious complications. Furthermore, in the multivariable analysis, empirical or definitive combination therapy did not appear to offer any mortality benefit over monotherapy for patients with *Pseudomonas* spp. hospital-acquired infections.

The results of this study are relevant to physicians in hospital settings who attend patients with a wide variety of disease (e.g., medical and non-medical patients) and severity (e.g., bacteremia and sepsis/septic shock). However, our study has a number of mentionable limitations. Firstly, since we included various types of hospital-acquired infections caused by *P*. *aeruginosa* and not only isolates from sterile sites or monomicrobial infections, a significant proportion of isolates were polymicrobial infections, which can influence the treatment responses and patient outcomes. Secondly, because of the retrospective design, the clonality of the isolates and mechanisms of resistance were not investigated. In addition, since we did not test the susceptibility to aztreonam (due to the unavailability of the drug in Thailand) or phosphonic acid (because no breakpoints are given in the CLSI guidance), we have probably to some extent overestimated the prevalence of XDR-PA in this study. Lastly, as fewer patients were included than we originally calculated, the statistical power of our results might have been insufficient to detect risk factors with low odd ratios.

## Conclusions

To our knowledge, this is the first study focusing on the epidemiology and risk factors for XDR-PA infections in Thailand. Given the high mortality rate of patients with XDR-PA infections, timely and appropriate empirical treatment with colistin should be considered for critically ill patients when an XDR-PA infection is suspected.

## Supporting information

S1 Dataset (XLSX)
